# High-Q trenched aluminum coplanar resonators with an ultrasonic edge microcutting for superconducting quantum devices

**DOI:** 10.1038/s41598-023-42332-6

**Published:** 2023-09-20

**Authors:** E. V. Zikiy, A. I. Ivanov, N. S. Smirnov, D. O. Moskalev, V. I. Polozov, A. R. Matanin, E. I. Malevannaya, V. V. Echeistov, T. G. Konstantinova, I. A. Rodionov

**Affiliations:** 1 FMN Laboratory, Bauman Moscow State Technical University, Moscow, 105005 Russia; 2 Dukhov Automatics Research Institute (VNIIA), Moscow, 127055 Russia

**Keywords:** Qubits, Superconducting devices

## Abstract

Dielectric losses are one of the key factors limiting the coherence of superconducting qubits. The impact of materials and fabrication steps on dielectric losses can be evaluated using coplanar waveguide (CPW) microwave resonators. Here, we report on superconducting CPW microwave resonators with internal quality factors systematically exceeding 5 × 10^6^ at high powers and 2 × 10^6^ (with the best value of 4.4 × 10^6^) at low power. Such performance is demonstrated for 100-nm-thick aluminum resonators with 7–10.5 um center trace on high-resistivity silicon substrates commonly used in Josephson-junction based quantum circuit. We investigate internal quality factors of the resonators with both dry and wet aluminum etching, as well as deep and isotropic reactive ion etching of silicon substrate. Josephson junction compatible CPW resonators fabrication process with both airbridges and silicon substrate etching is proposed. Finally, we demonstrate the effect of airbridges’ positions and extra process steps on the overall dielectric losses. The best quality factors are obtained for the wet etched aluminum resonators and isotropically removed substrate with the proposed ultrasonic metal edge microcutting.

## Introduction

Superconducting CPW microwave resonators are the basic elements of superconducting circuits: quantum processors^1^, quantum-limited parametric amplifiers^2^, quantum memory^3^, photon detectors^4^, and artificial atoms^5^. There are many applications where resonators operating in a single-photon regime are characterized by a significant internal quality factor (Q_i_) decrease due to dielectric losses in bulk dielectrics and thin interfaces containing two-level systems (TLS)^6,7^. Dielectric losses directly affect the performance of superconducting devices, for example, the relaxation times of qubits^6,8^. CPW resonators internal quality factor at low microwave power (Qi_LP_) depends dominantly on dielectric losses in interfaces: metal-substrate (MS), metal-vacuum (MA) and substrate-vacuum (SA) interfaces^9,10^. It has been shown, that the MS interface is dominant^10^ and it is generally determined by the choice of metal deposition and substrate cleaning procedures^11^. High Qi_LP_ values approaching 2.0 × 10^6^ were obtained for TiN^9^ and NbTiN^12^ CPW resonators. However, thick metal films up to 300 nm and 750 nm respectively were used, which makes it harder to incorporate to qubit fabrication. The best Qi_LP_ reaching 2.0–3.0 × 10^6^ in case of 100 nm thick aluminum film were demonstrated^13^ for CPW resonators with large cross section dimensions (center trace/gap of 24 μm). A silicon substrate etching with Al resonators was implemented in Ref.^14^, but with 250 nm thick aluminum the best Qi_LP_ up to 1.8 × 10^6^ was achieved. The internal quality factor of CPW resonators can be increased using new materials compatible with aggressive treatment, thicker superconducting films and larger cross section dimensions leading to lower field intensity. However, it is very hard to integrate them into superconducting qubit circuits fabrication processes. Aluminum technology is still one of the leading platforms for superconducting qubits^15,16^, which requires base sub-150 nm thick Al layer^16–18^ with optimized cross section dimensions of resonators (center trace up to 10 μm^19^). Improving aluminum CPW resonators quality requires further technology investigation: ultra-high vacuum Al deposition^13^, advanced substrate cleaning^17^, substrate etching^9,12^, and etc.

In this paper, we report on high Qi_LP_ aluminum 100 nm thick resonators on etched silicon substrates compatible with superconducting qubits fabrication. We investigate Al metal and Si substrate etching, as well as post treatment steps, in order to reduce the loss on the MA and SA interfaces. Using the proposed technology, we demonstrate internal quality factors at low Qi_LP_ and high Qi_HP_ power exceeding 2.0 × 10^6^ and 5.0 × 10^6^ respectively for identical resonators at frequencies ranging from 4.0 to 5.0 GHz. It is fabricated using isotropic substrate etching of optimized cross section dimensions of resonators (10.5 μm center trace and 3.5 μm gap) with both airbridges and without them. The best internal quality factors obtained for the 2.91 GHz resonator are Qi_LP_ = 4.4 × 10^6^ and Qi_HP_ = 1.9 × 10^7^. We achieve it by introducing isotropic silicon substrate etching with subsequent ultrasonic resonators edge microcutting after aluminum wet etching.

After resonators patterning, we fabricate airbridges to suppress parasitic slotline modes^20^. In order to evaluated airbridges influence on Qi_LP_, we measured identical resonators without airbridges, with airbridges over feedline only, and over both resonators and feedline. Using the proposed technology, we are able to reach state-of-the-art internal quality factor at low power for aluminum CPW resonators^9,12–14,17,21^ compatible with superconducting qubit circuits fabrication process (Table 1).

**Table 1:** CPW resonators comparison; w is the resonator center trace width, gap is the gap between resonator center trace and the ground, f_0_ is the resonant frequency, Qc is the coupling quality factors between feedline and resonators, and Qi_LP_ are the internal quality factors at low power.

f_0_ (GHz)	W (um)	Gap (um)	Film	Thickness (nm)	Substrate	Substrate etching	Qc, × 10^5	Qi_LP_, × 10^5	References
5.50–6.0	**24.0**	**24.0**	Al	100	Si 100	–	–	20.0–30.0	^13^
2.75–6.41	12.0	5.0	NbTiN	**160, 300**	Si 100	+	7.0–10.0	10.0–20.0	^12^
5.0–6.0	**28.0**	**14.0**	TiN	**450, 750**	Si 100	+	–	20.0	^9^
–	–	–	Al	**250**	Si 100	+	1.5–50.0	18.0	^14^
4.50	–	–	Al	150	Si 100	–	3.4	8.0	^17^
5.20–5.60	–	–	Nb	–	Si	+	–	8.4–11.8	^21^
2.91–5.0	7.0/10.5	4.0/3.5	Al	100	Si 100	+	2.0–4.0	16.5–44.0	This work

CPW resonators comparison; w is the resonator center trace width, gap is the gap between resonator center trace and the ground, f_0_ is the resonant frequency, Qc is the coupling quality factors between feedline and resonators, and Qi_LP_ are the internal quality factors at low power.

Significant values are in bold.

## Experimental details

To evaluate the effects of the Al film and Si substrate etching, airbridges fabrication, additional ultrasonic microcutting on Qi_LP_ of the resonators, we fabricated quarter-wave resonators according to the frequency multiplexing scheme^22^ on 25 × 25 mm silicon substrates with further cutting to 5 × 10 mm chips. There are 12 resonators on each chip with frequencies ranging from 4.0 to 7.0 GHz for devices without substrate etching and 6 resonators with frequencies ranging from 2.5 to 5.0 GHz for devices with substrate etching. All the resonators were designed to have 50 Ohm impedance (center trace widths/gap): 7.0/4.0 μm for resonators without substrate etching and 10.5/3.5 μm for resonators with substrate etching. The widths of the etched resonators are corrected to take into account the change in the effective dielectric permittivity (ε_eff_^23^) during substrate etching. The coupling quality factor Qc was designed to be 3.0 × 10^5^, but the experimental values are in the range of 2 × 10^5^ to 4 × 10^5^ due to simulation and design issues (It is difficult to determine the mutual influence of a large number of resonators on the Q_C_ by 3D modeling). The typical error in the determination of Qi_LP_ is 14%, and Qi_HP_ is 4%. A script^18^ based on a conformal mapping method was used to evaluate Qc and impedance of the resonators. In order to eliminate frequency dependence, we selected and compared the internal quality factors of the resonators with frequencies ranging from 4.0 to 5.0 GHz only.

For airbridges influence evaluation we used two designs: the first one with 9 airbridges over the feedline only; the second one with both 9 airbridges over the feedline and 4 airbridges evenly spaced over each resonator, which should be enough to eliminate the slotline modes^20^. Optical images of the chips can be found in the supplementary materials.

**Figure 1:**
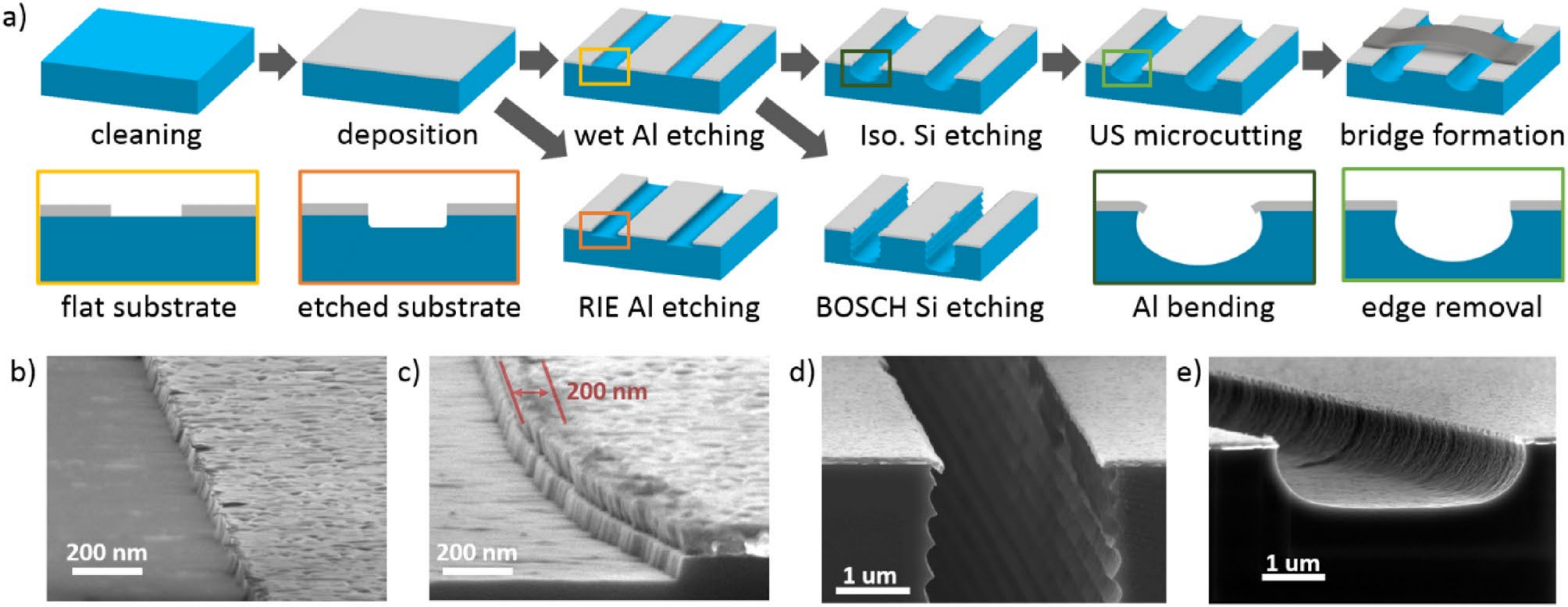
(**a**) Fabrication sequence of resonator chips with different Al film and Si substrate treatment. SEM images of the resonators center trace edges: (**b**) Al wet etching; (**c**) Al dry etching; (**d**) Si substrate Bosch DRIE; the sagging edges of the thin film can be observed; (**e**) Si substrate isotropic etching followed by ultrasonic microcutting.

Figure 1a shows the fabrication sequence scheme of resonator chips. We used high-resistivity Si(100) substrates (> 10kOhm-cm) for all the samples. Al films were deposited by ultrahigh-vacuum electron-beam deposition system under a base pressure lower than 10^–9^ Torr. Before deposition substrates were cleaned in RCA1 solution, followed by HF treatment to remove native oxide and terminate the Si surface with hydrogen. Then we installed Si substrates in the load lock as quickly as possible after cleaning, typically within 10 min. Al films with a thickness of 100 nm were deposited according to the regime used in Ref.^24^ and Ref.^25^ to form the base metal layer. After photoresist mask spincoating and patterning, Al films were etched either by wet etching in an industrial Aluminum Etching Type A solution (Fig. 1b) or by dry etching in a BCl_3_/Cl_2_ gas mixture (Fig. 1c). Then we dry etched the silicon substrate either by Bosch DRIE process^26^ with 90 cycles (Fig. 1d) or by isotropic RIE process in SF_6_ gas mixture. During Al etching process the edges of resonators center trace are usually damaged (Fig. 1b,c) by thermal or chemical influence. To remove these damaged metal edges, we optimize our substrate etching processes to get the desired undercut, then by using strong ultrasonic microcutting in isopropyl alcohol, we cut them to obtain high-quality metal edges (Fig. 1e). At the final stage, airbridges were formed for a group of resonators according to the technology used in superconducting qubit circuits fabrication^19^.

After dicing, the chips were mounted in copper sample holders made in according to the recommendations given in Ref.^27^, and mounted in a 10 mK stage in the dilution fridge. We used infrared and magnetic shielding to protect our samples against quasiparticles generations^28^ and magnetic vortices. We measured the transmission coefficient S_21_ of the resonators with a vector network analyzer (VNA) according to the method described in Ref.^29^. A total attenuation of 90 dB was installed on cryostat stages, all the measurements are performed under the temperatures 10 mK. The input and output lines were equipped with powder infrared filters-eccosorb, as well as low-pass filters. At the output line at 4 K stage, there is an amplifier on a high electron mobility transistor (HEMT). The wiring diagram of a measurement setup for the samples can be found in supplementary materials. We varied the drive power so that the photon population ⟨n_p_⟩ in the resonator ranged from the single-photon levels up to 10^7^ photons. We experimentally observed, that at the lowest power Qi_LP_ can fluctuate by more than 34% over several hours period due to fluctuations in TLS populations^30^. Here, we present the time-averaged Qi_LP_ values instead of maximum values.

## Experimental results and discussion

### Groups of resonators

**Figure 2:**
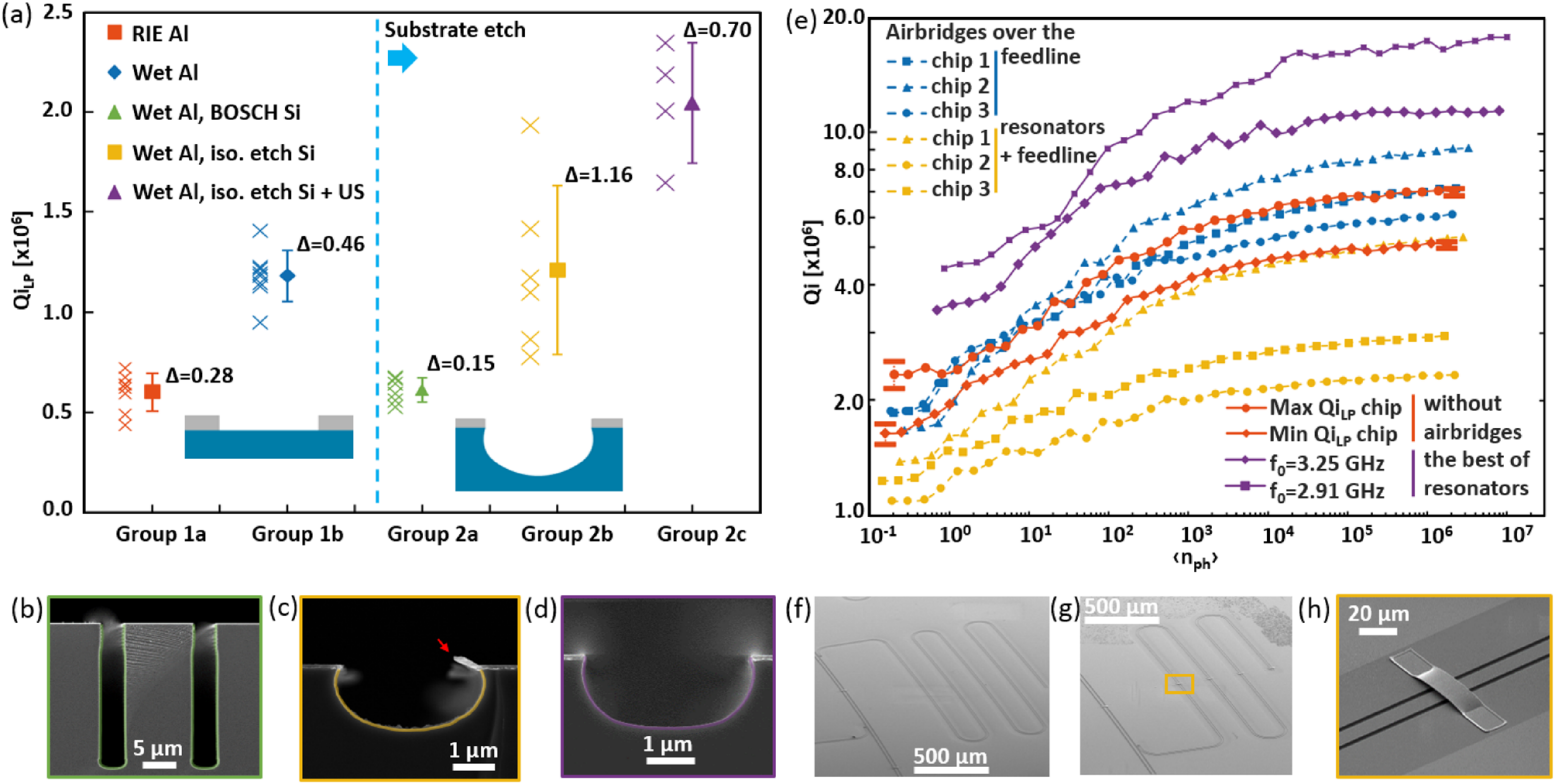
(**a**) Internal quality factor in single-photon regime of resonators grouped by fabrication technological features into groups. Group 1a—RIE Al, without substrate etching; group 1b—wet etching Al, without substrate etching; group 2a—wet etching Al, DRIE Bosch substrate; group 2b—wet etching Al, isotropic substrate etching; group 2c—wet etching Al, isotropic substrate etching, additional ultrasonic microcutting (crosses indicate the average value of Qi_LP_ for each resonator, whereas the error bars indicate the standard deviations and mean value). SEM images of the cross section of the resonators: (**b**) group 2a, (**c**) group 2b, (**d**) group 2c. (**e**) Intracavity photon number dependence of the internal quality factor of resonators with wet etching Al and isotropic etching of substrate with airbridges over feedline (blue lines), with airbridges over feedline and resonators (yellow lines), without airbridges (red lines, the bars show a typical error in determining the Qi) in 4—5 GHz range and outside this range (violet line) on average photon population in resonator. Lines were added for better visibility. (**f**) SEM image of the feedline section with bridges; (**g**) SEM image of the feedline and resonator section with bridges; (**h**) SEM image of a single bridge on resonator with etching of the substrate in the gap.

Figure 2a shows Qi_LP_ measurements for the CPW resonators grouped by different Al film and Si substrate etching technology. Groups 1a and 1b with the average Qi_LP_ of 6.0 × 10^5^ and 1.18 × 10^6^ include resonators obtained by RIE and wet Al etching, respectively, without Si substrate etching. Group 2a with the average Qi_LP_ of 6.1 × 10^5^ includes resonators obtained by wet Al etching with Si substrate Bosch DRIE. Groups 2b and 2c with the average Qi_LP_ of 1.21 × 10^6^ and 2.05 × 10^6^ contain resonators obtained by wet Al etching with Si substrate isotropic etching without ultrasonic edge microcutting and with it, respectively. Figure 2b–d show SEM images of the structure specifics for groups 2a, 2b, 2c. The measurement results of all our samples are shown in the supplementary materials.

### Resonators without substrate etching

One can notice the systematic dependence of Qi_LP_ on the metal and substrate etching processes. We found that the Qi_LP_ of resonators fabricated by wet etching is twice higher compared to our dry etching. We attribute this dependence to the metal-vacuum (MA) and substrate-vacuum (SA) interfaces having significantly lower loss tangents after wet etching than after dry etching. It could be definitely observed, that the surface of resonator center trace is damaged^31^ at a distance of about 200 nm from the edge (Fig. 1c), which is the area with the highest field intensity. At the same time, it was demonstrated by simulation^10^ that the substrate etching by only 10 nm reduces the participation ratio of the metal-air-substrate corners by 50%, while having a negligible impact on the other participation ratios, which should have a positive effect on the Qi_LP_ level. In our case, we have dry etched the substrate to 80 nm depth, but the Qi_LP_ level is still much lower than in the case of wet etching, where no etching of the substrate took place. We suppose that the reason is a very high concentration of TLS in the damaged region together with the high field intensity.

### Resonators with substrate etching

Bosch DRIE substrate etching allowed the fabrication of resonators with low Qi_LP_ values. The most possible reason is a high TLS concentration in the MA and SA interfaces as a result of incomplete removal of specific Bosch process polymer residues, which could be further cleaned. Isotropic etching of the Si substrate allowed a slight increase in Qi_LP_ compared to the level of wet-etched Al resonators (from 1.18 × 10^6^ to 1.21 × 10^6^), but the standard deviation in the group increased significantly. The possible reason is a non-reproducibility of metal edge geometry, which turns out to be "suspended" after etching, which negatively affects MA interface, resonator impedance and resonant frequency. We confirm this assumption by introducing an additional treatment in isopropyl alcohol with ultrasound: the "suspended" metal edge is broken off and the geometry of the resonators is reproduced exactly. With the width of the removed metal being of 600 nm, which is 3 times the width of the Al section damaged during etching, resulting in an almost twofold increase in the average Qi_LP_ to 2 × 10^6^ while the standard deviation value decreases.

### Resonators with substrate etching and airbridges

Figure 2e shows the Qi_LP_ dependences of resonators with wet Al etching and isotropic Si substrate etching with airbridges over the feedline (Fig. 2f,h, blue lines), with airbridges over the feedline and resonators (Fig. 2g, yellow lines), and without airbridges (red lines) on the average photon population in the resonator. The average photons number was determined based on applied power, Qc and the loaded quality factor Ql according to the recommendations from Ref.^32^. For the group of resonators without airbridges, only the results with the highest and lowest Qi_LP_ are shown. Figure 2e also shows Qi of our best resonators without air bridges at the frequencies 2.91 GHz and 3.25 GHz and Qi_LP_ equal to 4.4 × 10^6^ and 3.4 × 10^6^, respectively (violet line). One can notice, that airbridges location directly affects resonator Qi_LP_ (Fig. 2e), which is in a good agreement with Ref. ^20^. The airbridges placed over the feedline does not affect the resonators internal quality factor (it is within the variation of Qi_LP_ for resonators without airbridges).

## Conclusions

In summary, we have measured the internal quality factors of 100 nm thick aluminum CPW resonators which are compatible with superconducting qubits fabrication route for various base metal and silicon substrate etching processes, as well as post treatment technological step. Wet Al film etching with isotropic Si substrate dry etching followed by the proposed ultrasonic resonators edge microcutting leads to the average Qi_LP_ above 2.0 × 10^6^, achieved resonators with w = 10.5 μm and f_0_ = 4.0–5.0 GHz. The highest achieved Qi_LP_ value is 4.4 × 10^6^ for the resonator with w = 10.5 μm and f_0_ = 2.91 GHz. Finaly, we fabricate high quality factor superconducting CPW resonators with Si substrate etching and airbridges showing that the additional fabrication steps do not result in overall circuit performance decrease. The samples are fabricated at the BMSTU Nanofabrication Facility (Functional Micro/Nanosystems, FMNS REC, ID 74,300).

See supplementary material for the design and fabrication details for the two types of devices we investigated: a resonator circuit without substrate etching and a resonator circuit with substrate etching, measurment setup and wiring diagram of a measurement setup for the samples, table with all device fabrication parameters.

### Supplementary Information

Supplementary Information.

## Data Availability

The data that support the findings of this study are available within the article and its supplementary material.
