# Supplementary material for: High-Q trenched aluminum coplanar resonators with an ultrasonic edge microcutting for superconducting quantum devices
Source: Sci Rep. 2023 Sep 20;13:15536. doi: 10.1038/s41598-023-42332-6 (PMC10511541; doi:10.1038/s41598-023-42332-6)
Supplement: Supplementary file 1 — Supplementary Information. [file 41598_2023_42332_MOESM1_ESM.docx]

**Supplement to: High-Q trenched aluminum coplanar resonators with an ultrasonic edge microcutting for superconducting quantum devices**

E.V. Zikiy,^1,2^ A.I. Ivanov,^1,2^ N.S. Smirnov,^1,2^ D.O. Moskalev,^1,2^
V.I. Polozov,^1^ A.R. Matanin,^1,2^ E.I. Malevannaya,^1^ V.V. Echeistov,^1^
T.G. Konstantinova^1^ and I.A. Rodionov^1,2^

*^1^FMN Laboratory, Bauman Moscow State Technical University, Moscow 105005, Russia*

*^2^Dukhov Automatics Research Institute (VNIIA), Moscow 127055, Russia*

This supplement provides experimental details and data sets to support the claims made in the main text. First, we present the design and fabrication details for the two types of devices we investigated: a resonator circuit without substrate etching and a resonator circuit with substrate etching. We then describe a several technical details of the measurement system: the measurement setup and device shielding and the fitting of the complex-valued transmission spectra. Next, we present the measurement results for the type 1 and type 2 devices.

**DEVICES**


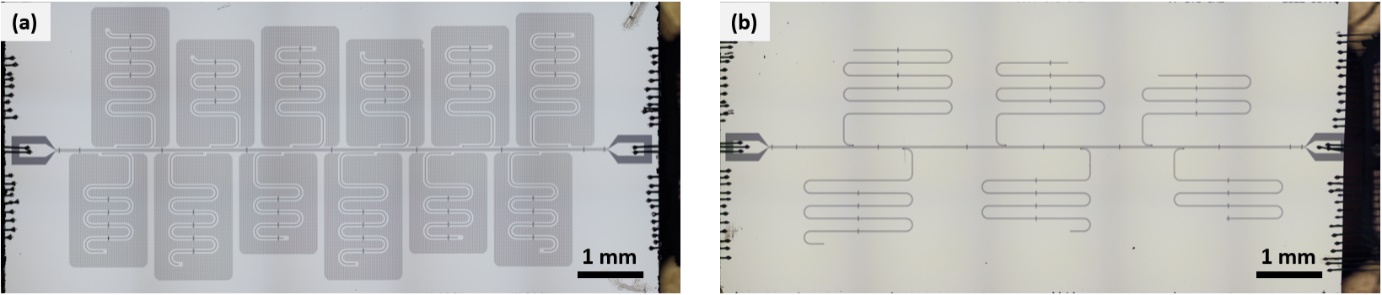


FIG. S1: (a) Optical image of a 12-resonators circuit with frequencies from 4.0 to 7.0 GHz without substrate etching (type 1). (b) Optical image of a 6-resonators circuit with frequencies from 2.5 to 5.0 GHz with isotropic substrate etching (type 2). Both resonator circuits are fabricated using wet etching in an industrial Aluminum Etching Type A solution. Resonator circuits with airbridges over feedline and resonators are presented.

On the chip without substrate etching (Fig. S1(a)) there are 12 resonators with frequencies: 4.0, 4.4, 4.6, 4.8, 5.0, 5.4, 5.6, 6.0, 6.4, 6.6, 6.8, 7.0 GHz and on the chip with isotropic substrate etching (Fig. S1(b)) there are 6 resonators with frequencies: 2.5, 3.0, 3.5, 4.0, 4.5, 5.0 GHz. For silicon isotropic etching we use hight‑density plasma etching at RF power of 700 W and bias power of 40 W, a SF_6_ with 35 sccm flow rates, and 40 mTorr pressure. Table S1 shows the fabrication parameters of all devices presented in this work.

**MEASUREMENT SETUP**

To minimize resonator losses induced by non-equilibrium quasiparticles and magnetic vortex displacement, we mount the samples inside several layers of shielding (Fig. S2). Specifically, we anchor the PCB-mounted sample directly to a copper cold finger connected to the mixing chamber of the dilution refrigerator. The sample is then enclosed in a copper can, the inner surface of which is coated in a mixture of Stycast 2850 FT and silicon carbide granules with diameter 1000 µm. This can is enclosed in a second aluminum can. This is finally enclosed in one layer of cryogenic magnetic shielding (1-mm-thick Cryoperm). Coaxial cables entering the sample holder were pasted into the lid of the inner layer of IR shielding to reduce the impact of open holes on the shielding effectiveness. Extra radiation shielding is provided by in-house inline Eccosorb infrared filters in the input coaxial line mounted outside the magnetic shields at the mixing chamber stage.


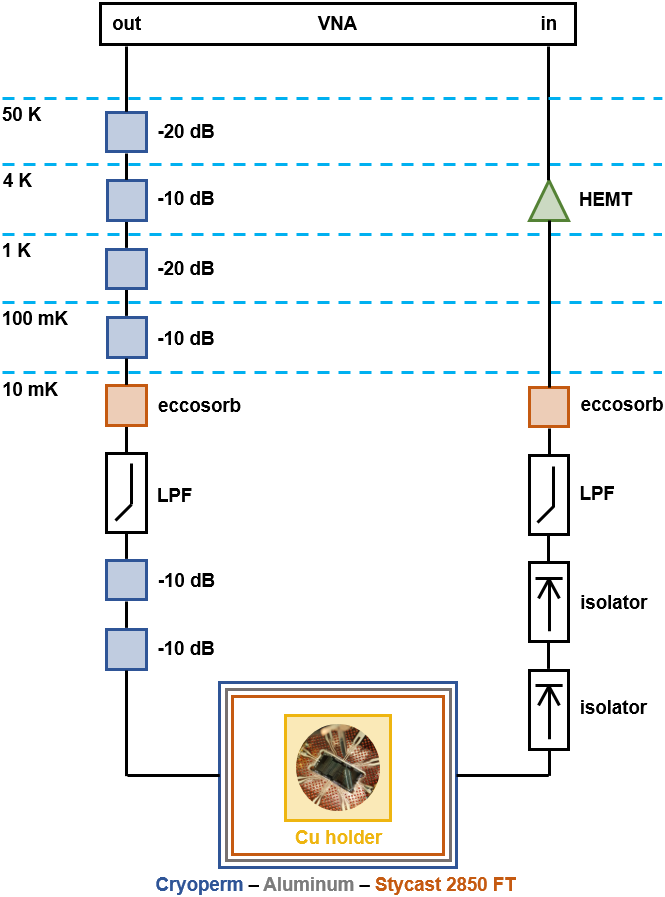


FIG. S2: Wiring diagram of a measurement setup for the samples

All measurements are performed by means of a vector network analyzer (VNA) Rohde&Schwarz ZNB20 in the hanger mode [1]. The typical S_21_ magnitude response of superconducting resonator is shown in Figure S3.

The measurement procedure performs S_21_ magnitude response measurement in the frequency range of 200-500 kHz around the resonance for each resonator. The resonance peak is located in the center of chosen range. We perform sweep routine of complex S21 measurements response from low-powers to the high power and extract the resonant frequency *f_res_*, loaded quality factor Q_l_, coupling quality factor Q_c_ and internal quality factor Q_i_ using opensource package described in detail in [2]. The power limits are chosen experimentally, so that the low-power corresponds to the single-photon regime, and the high-power does not put the resonator into nonlinear regime. The typical Q-factor profiles are shown in FIG.2 in the main text.


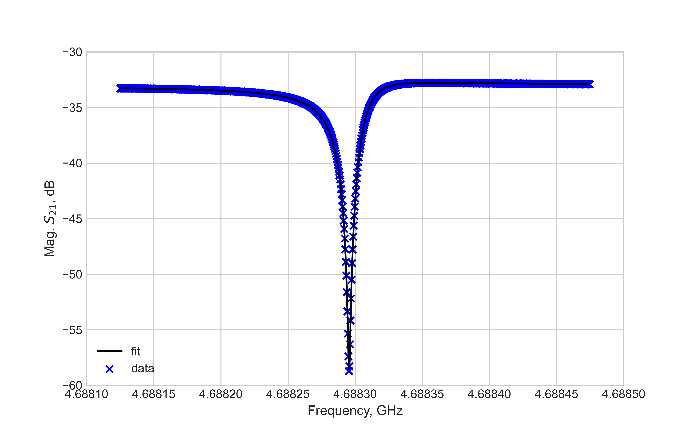


FIG. S3: Typical S_21_ magnitude response of superconducting resonator. Dashed line is data points, solid line is a fit from which we extract the resonator Q-factors (device 2b.3 in table S1)

1. Gao, J. et. al. “Experimental evidence for a surface distribution of two-level systems in superconducting lithographed microwave resonators”

2. S. Probst, F. Song, P. Bushev, A. Ustinov, and M. Weides, Review of Scientific Instruments 86, 024706 (2015)

**TLS LOSS**

The TLS-based loss model is known [1]:

$\frac{\boldsymbol{1}}{\boldsymbol{Q}_{\boldsymbol{i}}}\boldsymbol{=F}\boldsymbol{\delta}_{\boldsymbol{TLS}}^{\boldsymbol{0}}\frac{\tanh\boldsymbol{(}\frac{\boldsymbol{\hbar}\boldsymbol{\omega}_{\boldsymbol{0}}}{\boldsymbol{2}\boldsymbol{k}_{\boldsymbol{B}}\boldsymbol{T}}\boldsymbol{)}}{{\boldsymbol{(1+}\left( \frac{\left\langle\boldsymbol{n} \right\rangle}{\boldsymbol{n}_{\boldsymbol{c}}} \right)\boldsymbol{)}}^{\boldsymbol{\beta}}}\boldsymbol{+}\boldsymbol{\delta}_{\boldsymbol{other}}$ **(1)**

A fit to Eq. (1) for resonator 2c.2 is shown in Fig. S4. This shows F=1.05, n_C_=2.99, β=3.36 x10^-1^, δ_other_ = 1.38 x10^-7^.


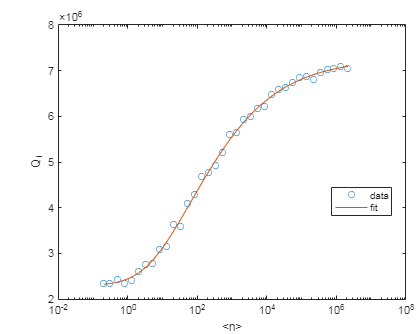


FIG. S4: Plot of Qi as a function of <n> for the resonator at 10 mK. In orange is a fit to TLS

losses described by Eq. (1)

1. Burnett, J., Bengtsson, A., Niepce, D., & Bylander, J. (2018, March). Noise and loss of superconducting aluminium resonators at single photon energies. In *Journal of Physics: Conference Series* (Vol. 969, No. 1, p. 012131). IOP Publishing.

TABLE S1. Device fabrication parameters

| device | f, GHz | Qi_LP_x10^6^ | Qi_HP_x10^6^ | wet etch Al | RIE Al | Bosch Si | iso. etch Si | US microcutting | feedline bridges | resonator bridges |
| --- | --- | --- | --- | --- | --- | --- | --- | --- | --- | --- |
| 1a.1 | 4.45 | 0.718 | 2.739 |  | x |  |  |  |  |  |
| 1a.2 | 4.55 | 0.619 | 2.614 |  | x |  |  |  |  |  |
| 1a.3 | 4.84 | 0.674 | 5.292 |  | x |  |  |  |  |  |
| 1a.4 | 4.45 | 0.435 | 2.206 |  | x |  |  |  |  |  |
| 1a.5 | 4.55 | 0.591 | 3.588 |  | x |  |  |  |  |  |
| 1a.6 | 4.94 | 0.490 | 3.089 |  | x |  |  |  |  |  |
| 1a.7 | 4.44 | 0.638 | 5.852 |  | x |  |  |  |  |  |
| 1a.8 | 4.53 | 0.634 | 5.614 |  | x |  |  |  |  |  |
| 1b.1 | 4.40 | 1.193 | 4.864 | x |  |  |  |  |  |  |
| 1b.2 | 4.40 | 1.226 | 4.797 | x |  |  |  |  |  |  |
| 1b.3 | 4.60 | 0.947 | 0.947 | x |  |  |  |  |  |  |
| 1b.4 | 4.60 | 1.215 | 3.059 | x |  |  |  |  |  |  |
| 1b.5 | 4.40 | 1.406 | 5.095 | x |  |  |  |  |  |  |
| 1b.6 | 4.60 | 1.128 | 4.395 | x |  |  |  |  |  |  |
| 1b.7 | 4.82 | 1.182 | 3.646 | x |  |  |  |  |  |  |
| 1b.8 | 4.46 | 1.144 | 6.032 | x |  |  |  |  |  |  |
| 2a.1 | 4.56 | 0.667 | 2.623 | x |  | x |  |  |  |  |
| 2a.2 | 4.55 | 0.593 | 4.122 | x |  | x |  |  |  |  |
| 2b.1 | 4.39 | 0.862 | 2.648 | x |  |  | x |  |  |  |
| 2b.2 | 4.02 | 0.776 | 2.791 | x |  |  | x |  |  |  |
| 2b.3 | 4.69 | 1.417 | 2.945 | x |  |  | x |  |  |  |
| 2a.3 | 4.55 | 0.525 | 3.569 | x |  | x |  | x |  |  |
| 2a.4 | 4.04 | 0.673 | 3.887 | x |  | x |  | x |  |  |
| 2a.5 | 4.64 | 0.641 | 3.861 | x |  | x |  | x |  |  |
| 2a.6 | 4.04 | 0.557 | 5.367 | x |  | x |  | x |  |  |
| 2b.4 | 4.07 | 1.933 | 6.117 | x |  |  | x |  |  |  |
| 2b.5 | 4.32 | 1.097 | 2.251 | x |  |  | x |  |  |  |
| 2b.6 | 4.07 | 1.172 | 3.816 | x |  |  | x |  |  |  |
| 2c.1 | 4.33 | 2.005 | 5.820 | x |  |  | x | x |  |  |
| 2c.2 | 4.36 | 2.346 | 7.100 | x |  |  | x | x |  |  |
| 2c.3 | 4.72 | 1.645 | 5.169 | x |  |  | x | x |  |  |
| 2c.4 | 4.00 | 2.184 | 10.391 | x |  |  | x | x |  |  |
| 2c.5 | 2.91 | 4.447 | 18.297 | x |  |  | x | x |  |  |
| 2c.6 | 3.25 | 3.457 | 13.407 | x |  |  | x | x |  |  |
| 2c.7 | 4.39 | 1.861 | 7.203 | x |  |  | x | x | x |  |
| 2c.8 | 4.02 | 1.680 | 9.176 | x |  |  | x | x | x |  |
| 2c.9 | 4.76 | 1.884 | 6.159 | x |  |  | x | x | x |  |
| 2c.10 | 4.02 | 1.388 | 5.371 | x |  |  | x | x | x | x |
| 2c.11 | 4.39 | 1.095 | 2.335 | x |  |  | x | x | x | x |
| 2c.12 | 4.76 | 1.238 | 2.957 | x |  |  | x | x | x | x |
